# Evolution of novel genes in three-spined stickleback populations

**DOI:** 10.1038/s41437-020-0319-7

**Published:** 2020-06-04

**Authors:** Jonathan F. Schmitz, Frédéric J. J. Chain, Erich Bornberg-Bauer

**Affiliations:** 1 Institute for Evolution and Biodiversity, University of Münster, Münster, Germany; 2 Department of Biological Sciences, University of Massachusetts, Lowell, MA USA

**Keywords:** Evolutionary genetics, Molecular evolution, Population genetics

## Abstract

Eukaryotic genomes frequently acquire new protein-coding genes which may significantly impact an organism’s fitness. Novel genes can be created, for example, by duplication of large genomic regions or de novo, from previously non-coding DNA. Either way, creation of a novel transcript is an essential early step during novel gene emergence. Most studies on the gain-and-loss dynamics of novel genes so far have compared genomes between species, constraining analyses to genes that have remained fixed over long time scales. However, the importance of novel genes for rapid adaptation among populations has recently been shown. Therefore, since little is known about the evolutionary dynamics of transcripts across natural populations, we here study transcriptomes from several tissues and nine geographically distinct populations of an ecological model species, the three-spined stickleback. Our findings suggest that novel genes typically start out as transcripts with low expression and high tissue specificity. Early expression regulation appears to be mediated by gene-body methylation. Although most new and narrowly expressed genes are rapidly lost, those that survive and subsequently spread through populations tend to gain broader and higher expression levels. The properties of the encoded proteins, such as disorder and aggregation propensity, hardly change. Correspondingly, young novel genes are not preferentially under positive selection but older novel genes more often overlap with F_ST_ outlier regions. Taken together, expression of the surviving novel genes is rapidly regulated, probably via epigenetic mechanisms, while structural properties of encoded proteins are non-debilitating and might only change much later.

## Introduction

Several studies over the last decades have demonstrated that genomes evolve rapidly, generating abundant genetic diversity at the level of populations (Zhao et al. 2014; Durand et al. 2019; Witt et al. 2019). Gene content and gene order can strongly differ between populations and even between individuals within populations.

For a long time, gene duplication was seen as the only important mode of gene emergence (Long et al. 2003). Gene duplication is an attractive model as it immediately explains the functional potential of the novel sequence. The process often starts with the duplication of a DNA sequence. Such genomic rearrangements first appear as copy number variations (CNVs) at the population level. CNVs are the result of duplications or deletions of genomic regions among individuals that can include the duplication (or multiplication) of genes (Katju and Bergthorsson 2013; Chain et al. 2014). On a short time scale, gene duplications (emerging as CNVs in populations) can lead to expression changes that have an adaptive benefit. Indeed, differences in gene copy numbers between populations can lead to differential gene expression consistent with local adaptation (Huang et al. 2019). Such fitness advantage, however, is not the most prevalent consequence of duplication, but rather expression attenuation of either of the gene copies, or gene silencing and loss (Tautz and Domazet-Lošo 2011; De Smet et al. 2013). Expression changes, foremost attenuation, have been attributed to either *cis*-regulatory changes (Huang et al. 2019) or epigenetic regulation (Keller and Yi 2014; Wang et al. 2017), such as gene-body methylation.

In addition to the duplication of existing genes, novel genes can also emerge from non-coding sequences, i.e., de novo. The definition of de novo sensu stricto only includes genisation of intergenic sequences. However, novel genes can also emerge from non-coding genic sequences such as introns (Ruiz-Orera et al. 2015; Prabh and Rödelsperger 2019). The starting point of this process is frequently the spurious expression of large intergenic regions in eukaryotic genomes at low levels (Durand et al. 2019; Witt et al. 2019; Carvunis et al. 2012; Neme and Tautz 2016; Nagalakshmi et al. 2008; Kapranov and Laurent 2012). While most intergenic transcripts do not have any significant function, i.e. one that is selected for or physiologically beneficial, some transcripts have been shown to become fixed and overlap with novel ORFs, which are also randomly acquired (Ruiz-Orera et al. 2014; Schmitz et al. 2018). As a consequence, some transcripts are prone to become either functional RNAs (Heinen et al. 2009; Mercer et al. 2009) or, if translated as well, they become expressed as proteins. Indeed, many transcripts also contain ORFs that are translated into short proteins (Wilson and Masel 2011; Vanderperre et al. 2013), thus exposing the encoded ORFs to selection (Chen et al. 2015; Xie et al. 2012; Zhang et al. 2019). It is to date unclear how often this transition from intergenic to expressed non-coding and further to coding sequences (i.e., de novo gene emergence) happens. In addition, the relative prevalence of transcription first vs. ORF first in this process has not been determined yet. One recent study proposes de novo emergence to be the dominant mechanism of novel gene emergence (Vakirlis et al. 2019). In some cases, de novo genes can emerge and contribute to increased fitness, as they can soon become essential (Zhang et al. 2019), e.g., for reproduction (Gubala et al. 2017). In the case of gene duplication, novel transcripts emerge from duplicated genes that diverge beyond the limits of homology detection. During de novo gene birth, transcripts emerge from non-coding sequences that as such do not have homologous sequences amongst protein-coding genes. In either case, rapid loss of de novo or duplicated “novel” genes is the most likely immediate outcome (Tautz and Domazet-Lošo 2011).

Other proposed mechanisms of how novel, though not strictly de novo, genes are created include the alternative (same of complementary strand) transcription of a CDS (Prabh and Rödelsperger 2019; Van Oss and Carvunis 2019), overprinting (i.e., the reuse of an existing ORF in an alternative reading frame) (Sabath et al. 2012) or the partial extension of reading frames (Bornberg-Bauer et al. 2015; Klasberg et al. 2018; Toll-Riera and Albà 2013).

Transcripts offer a valuable insight into gene emergence as they can be easily verified (in comparison to proteome studies) and do not rely on the difficult processes of gene annotation, which often also relies on homology signals. Consequently, studying novel transcripts allows analysis of the earliest stages of new gene emergence. Only a small fraction of transcripts is expected to survive the purifying effects of selection and eventually become fixed as a new gene. While the term “gene” itself is undergoing frequent semantic changes (Gerstein et al. 2007; Keeling et al. 2019), we here refer to it as a genetic segment transcribing a transcript for which evidence of translation exists, preferably but not necessarily experimentally verified.

New genes have repeatedly been suggested to provide strong adaptive benefits, especially in an ecological context (Chain et al. 2014; Zhang et al. 2019; Khalturin et al. 2009; Kumar et al. 2015). Accordingly, the early stages of gene emergence are of particular interest and need to be further investigated at the level of populations. This is especially important considering that most research on novel genes in general and de novo genes in particular remains controversial. First, signals of the selection of the encoded proteins have been observed in most (Chen et al. 2015; Zhang et al. 2019; Gubala et al. 2017; Palmieri et al. 2014), but not all (Guerzoni and McLysaght 2016) studies. Second, these proteins have been claimed to undergo rapid changes in structural properties which are deemed essential for functioning such as aggregation propensities, size and disorder content in some (Palmieri et al. 2014; Wilson et al. 2017), but not in other studies (Schmitz et al. 2018). In addition, all studies so far have compared genomes of different species and therefore consider evolution over relatively long time scales (i.e., several millions of years and longer).

Another understudied area is the effect and regulation of gene emergence in natural populations. So far, most studies on expression—and all focusing on novel genes—have been conducted in model species (Zhao et al. 2014). Most model species have not been under natural selection for many generations and may be subject to adaptation to laboratory conditions. A well-suited ecological model species is the three-spined stickleback, which is known to undergo rapid adaptation to many environmental conditions across the northern hemisphere (Foster and Bell 1994; McKinnon and Rundle 2002). This coastal fish has extensive genomic (Jones et al. 2012; Roesti et al. 2013; Glazer et al. 2015; Feulner et al. 2013, 2015) and transcriptomic data available (Feulner et al. 2013; Huang et al. 2016; Hanson et al. 2017; Metzger and Schulte 2018) and recent studies have demonstrated patterns of differences in CNVs between populations (Chain et al. 2014; Huang et al. 2019; Hirase et al. 2014), which make the system amenable to study the emergence and disappearance of novel genes.

Here, we take advantage of the genomic and transcriptomic datasets available for three-spined sticklebacks to study the distribution of transcripts between populations. Accordingly, we analysed the emergence and spread of new genes expressed in four tissues across nine populations of the three-spined stickleback. Gene expression levels and tissue specificity were compared across genes of different ages, finding older genes to be expressed more strongly and broadly. We also analysed the overlap of genes with CNVs and found younger new genes to overlap with CNVs more often than older new genes, showing how genomic changes facilitate new gene emergence in populations.

## Results and discussion

**Fig. 1:**
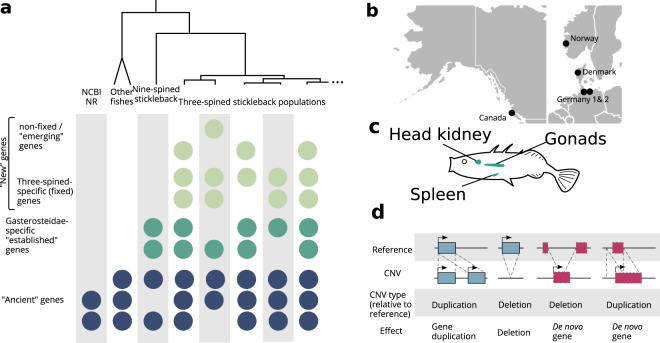
Illustrations of the gene age and visual presentation of data sources. **a** Examples for how genes of different ages are distributed across the analysed species. Each circle represents one gene being present in one species. Each gene is displayed on a separate row. Genes in the same row represent orthologs. Colours are used to distinguish age classes. **b** Locations of the population pairs the data was sampled from. **c** Visual representation of the organs sampled. **d** Pictogram depicting how CNV events can lead to gene duplication, deletion or de novo gene emergence.

Transcriptomes were sampled from multiple stickleback individuals from nine different populations taken from lake, river, and marine ecosystems in Europe and North America (as described in Chain et al. 2014 and Feulner et al. 2013, 2015, see also Supplementary Table 1 and Fig. 1). Four tissues were included in the analysis: the head kidney and spleen, as major immunity-associated tissues, and ovaries and testes, as reproductive tissues. Overall, after removing low-quality or outlier transcriptomes, we made use of 93 transcriptomes. The sampling design thus allows to analyse expressed sequences across an array of tissues and along a phylogeographic gradient (see Fig. 1 and Chain et al. 2014 for details). This analysis allows us to closely study the emergence of novel sequences.

To relate the properties of genes to their age, stickleback genes were grouped into one of three age groups. First, all genes with BLASTP hits with an E-value below 10^−3^ outside of *Gasterosteidae* or Pfam domains were categorised as “ancient” (see also Supplementary Fig. S1). For this homology search we used the NCBI NR database. Second, genes with TBLASTN hits in the nine-spined stickleback’s transcriptome were categorised as “established”, because they exist in at least two stickleback species and are *Gasterosteidae* specific. For this purpose we used the nine-spined stickleback’s transcriptome from the available brain and liver samples (Guo et al. 2013). Genes without hits in any of these homology searches represent recently emerged novel genes and where categorised as “new”. If they were present in the genome and all populations (at least once), i.e., they were sub-categorised as “fixed”, whereas they were sub-categorised as “emerging” if the transcripts were found in fewer populations. Clearly the line between “fixed” and “emerging” is blurred, because some transcripts might not have been observed in all populations due to low expression under the sampled natural conditions. However, we expect this to be minimal since we include several individual transcriptomes per population and apply a relatively stringent and widely used minimum threshold to consider a transcript as expressed (see “Methods” section). We found 11,413 ancient, 245 established and 991 new genes being expressed across all of our transcriptomes. More than half of the ancient genes are annotated, while only a small fraction (<5%) of the established and new genes is annotated in the three-spined stickleback’s Ensembl annotation (Supplementary Fig. S3).

### Younger genes are expressed less broadly and at lower levels

**Fig. 2:**
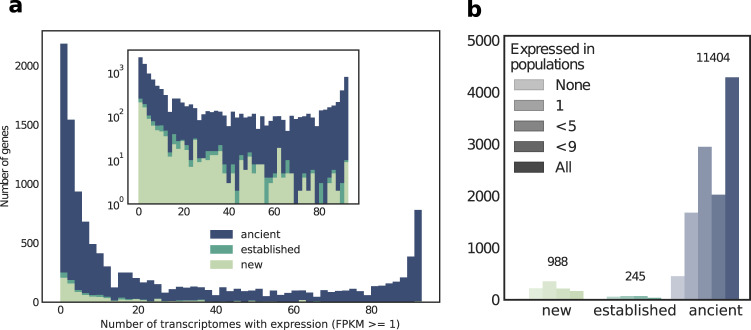
Distribution of transcript frequencies across transcriptomes and populations. **a** Number of transcriptomes each gene was found to be transcribed in shown as a stacked bar plot. Inset: same plot with log-scaled *y*-axis. Genes with expression levels >1FPKM were counted as expressed. **b** Numbers of genes found to be expressed in varying numbers of populations.

To assess the expression patterns of new genes, we analysed the distribution of their expression across all 93 transcriptomes (Fig. 2a). Out of the 12,637 genes we found expressed in the three-spined stickleback, 2187 (17%) were not found to be transcribed at a level above 1 FPKM (fragments per kilobase of transcript per million mapped reads) in more than one of the transcriptomes. Such low expression levels cannot be properly distinguished from transcriptional noise and were counted as not expressed (Zhao et al. 2014; Chen et al. 2015; Ramsköld et al. 2009). On the other hand, many—especially the “ancient” genes—are expressed above 1 FPKM in more than 90% of the transcriptomes (1708 genes, 14%). In comparison, only 2% of the “new” and “established” genes are found in more than 90% of all the samples, significantly fewer than ancient genes (*p* < 10^−30^, chi-square).

Among the nine populations, more than one-third (38%) of all “ancient” genes are found to be expressed in all populations, while only 17% of “new” genes are found in all populations (*p* < 10^−37^, chi-square; Fig. 2b, c). These findings show that younger genes are more often restricted to fewer transcriptomes and are population specific in their expression. This finding also supports previous findings on the unstable transcription of new genes (Li et al. 2019). One caveat here is that we only surveyed expression in four tissues. Consequently, it is possible that new genes are expressed in further populations but were not picked up. This question should be addressed in future, even broader studies.

**Fig. 3:**
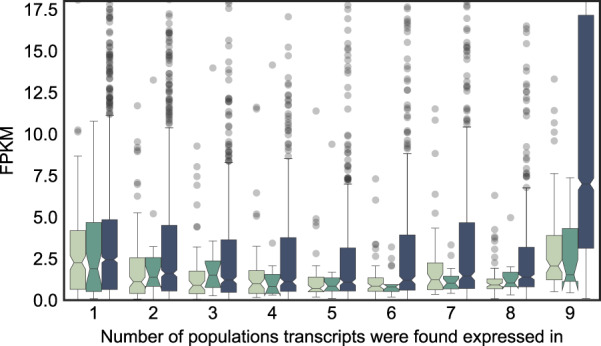
Box plot of expression strength (in FPKM) of genes by the number of populations the gene was found expressed in. Outliers beyond 17.5 are not shown.

We also evaluated the expression level of genes according to their age and prevalence among populations to analyse how new emerging genes spread through populations (Fig. 3a). We find that younger genes generally exhibit lower levels of expression than older genes. “New” genes show the lowest average expression levels, followed by “established” genes with intermediate expression levels, while “ancient” genes have the highest expression levels (*p* < 10^−56^). We also found highly similar results for expression specificity, with older genes being more broadly expressed compared with younger genes (Supplementary Fig. S6).

Our findings show that most new genes at first gain low and unstable, i.e. variable between individuals and populations, levels of transcription in single populations (Fig. 3). Such unstable expression is likely to be lost quickly again in a turnover of transcription (Neme and Tautz 2016). Over time, some of the “new” genes appear to spread through populations, i.e., “established” and “ancient” genes are expressed in more populations than “new” genes. This spread occurs in a process that is either biased towards new genes with already high expression levels, or that leads to an increase in expression levels and breadth. These findings are in line with previous studies that have found a high turnover of spurious transcription shortly after de novo gene emergence (Neme and Tautz 2016), and that new genes are spread gradually through populations (Zhao et al. 2014).

Gene-body methylation is a mechanism that could act to rapidly regulate the expression patterns of newly emerged genes (Zemach et al. 2010), similarly to how duplicate genes are regulated (Wang et al. 2015). Gene-body methylation has been shown to be a rapid mechanism of adaptation of expression strength, enabling adaptation to ecological factors (Rando and Verstrepen 2007; Huang et al. 2017). We find the two younger gene classes (established and new) to be less CpG depleted compared with older genes (Wilcoxon rank sum tests: *p* < 10^−7^). Established genes show the strongest CpG depletion. All genes have lower CpG enrichment than intergenic sequences (Supplementary Fig. S4). These findings show that gene-body methylation could play an important role in novel gene fixation, for example, as a first, fast way of regulation after new gene emergence.

### Younger genes are more often gonad specific

**Fig. 4:**
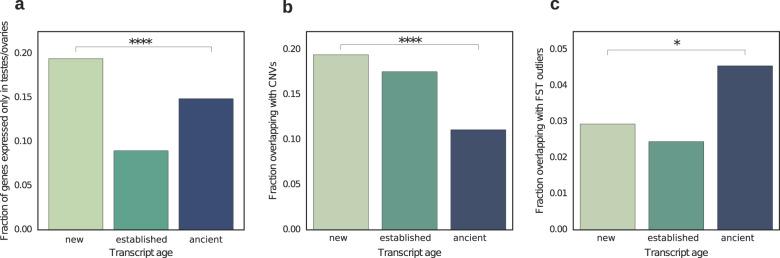
Differences in gene properties across age classes. **a** Fraction of genes expressed only in testes or ovaries. The *p* value shown here is the result of a 2 × 2 chi-square test. **b** Fraction of genes overlapping with CNVs. The *p* value shown here is the result of a 2 × 2 chi-square test between the “new” and “ancient” classes. ****p < 0.0001. **c** Fraction of genes overlapping with regions with F_ST_ indicating positive selection. The *p* value shown here is the result of a 2 × 2 chi-square test between the “new” and “ancient” classes. *p < 0.05.

Our results show that new genes are expressed at lower levels. Previously, new (duplicated and novel) genes have also been suggested to often be gonad biased (Kaessmann 2010; Cui et al. 2014). However, the expression levels of new genes have previously not been tested at a population level. Here, we compared the number of gonad-specific genes between age groups (Fig. 4a) to study the long suspected over-representation of testis bias in new gene emergence. Consistent with previous reports (Cui et al. 2014; Wu et al. 2014; Tobler et al. 2017), we find that “new” genes are 30% more likely to be expressed only in testes or ovaries compared with “ancient” genes (*p* < 0.001, 2 × 2 chi-square test). One could expect this bias to be caused by a lower number of reads required to reach the FPKM threshold to be counted as expressed, however, this is not the case here (see Supplementary Fig. S7).

This gonad specificity of new and established genes aligns with the hypothesis that gonads play an important role in new gene emergence (Kaessmann 2010; Cui et al. 2014). For example, in *Xenopus tropicalis*, young duplicated genes and novel genes are predominantly expressed in one sex and one gonad (Chain 2015). Also, recent findings showing reproductive functions of novel (including some de novo) genes in flies (Gubala et al. 2017; Reinhardt 2013; Kondo et al. 2017) suggest that novel genes often affect reproductive functions. These findings could explain why novel genes are more often expressed specifically in gonads, although it remains unclear whether this trend is causing the out-of-testis effect or caused by it. Furthermore, we also find “new” genes to show a more tissue-specific expression pattern than older genes (Fig. S6).

### Younger genes more frequently overlap with structural variations

To study the possible role of CNVs in the emergence of new genes, we compared the fraction of genes originating from genomic regions with duplications and deletions (CNVs) among three-spined stickleback individuals using matched genomes and transcriptomes. Recently, using the same data set, young duplicated genes were shown to evolve rapidly and potentially be involved in local adaptation in sticklebacks (Chain et al. 2014; Huang et al. 2019). Here, we find that “new” and “established” genes more often overlap with CNV regions (17% and 20% of “new” and “established” genes, respectively) compared with older genes (10%; *p* < 0.0001; Fig. 4b).

New genes might have a higher likelihood to overlap with CNVs because most new genes emerge through mutations that are observable as CNVs (see Fig. 1d). Consequently, some of the CNVs we observe might be the very CNVs that have caused the overlapping gene to emerge. De novo genes can emerge from the duplication of an existing, non-coding genomic sequence, if this duplication leads to the formation of a new ORF (Fig. 1d). Deletions can also lead to de novo gene emergence by creating new ORFs or causing existing ORFs to be transcribed (Fig. 1d).

In addition to CNVs, we determined the fraction of genes overlapping with regions exhibiting an increase in sequence differentiation (F_ST_, Fig. 4c). Here, we looked for instances of increased F_ST_ that have signatures of positive selection (from Feulner et al. 2015). In doing so, we found that older genes more often overlap with regions possessing high F_ST_ with signals of positive selection than younger genes. Consequently, positive selection acting on advantageous variants of older genes might more often be the cause of selective sweeps than selection acting on new genes. However, F_ST_ can only be calculated on regions present in all populations, i.e., regions without CNVs. Because new genes are more likely to overlap with CNVs, we also determined the fraction of genes overlapping F_ST_ outliers after excluding all genes in CNV regions, but the results were consistent whether we included or excluded CNVs.

### Protein properties show no difference with gene age

To determine whether the properties of novel proteins encoded by new genes differ from those of older proteins, we analysed the structural and sequence properties of all genes (see Supplementary Fig. S5). We apply IUPred (Dosztanyi et al. 2005) and TANGO (Fernandez-Escamilla et al. 2004) on the primary protein sequence of each gene to predict disorder and aggregation propensity, respectively. These programs are routinely used in comparable studies to allow for the sequence-based analysis of large datasets (Carvunis et al. 2012; Basile et al. 2017; Angyan et al. 2012). Both measures describe important properties of proteins. Protein disorder has significant functional influence because disordered protein regions can, e.g., represent flexible binding regions (Tompa 2011).

The avoidance of aggregation represents a critical force in protein evolution. Aggregating proteins are not functional and pose a fitness burden on the cell because they are toxic (Monsellier and Chiti 2007; Geiler-Samerotte et al. 2011). Consequently, new genes are expected to avoid aggregation and might rely on disordered regions for initial functions. Essentially, genes with high aggregation propensity are expected to lower fitness so much that they will not be observed in adult organism since they, e.g., disrupt developmental processes. Interestingly, the roles of both properties, disorder and aggregation, are controversially discussed in computational (Zhao et al. 2014; Carvunis et al. 2012; Basile et al. 2017) and experimental studies (Tretyachenko et al. 2017).

Here, we do not find structural properties to show measurable differences between any of the three age classes (Supplementary Fig. S2a, b). The properties of the nucleotide sequence, however, are different with the “ancient” genes exhibiting a significantly higher hexamer score and sequence length (Supplementary Fig. S5a, b). Similarly, the amino acid composition of “ancient” genes showed some difference to the two younger categories (Supplementary Fig. S8). These differences were found only between “new” and “ancient” genes. “Established” genes here closely resemble “new” genes, indicating that no adaptation of such sequence properties has taken place since these “established” genes have emerged. This, however, does not preclude further adaptation to take place on longer time scales, as is suggested by the higher chance of overlapping with F_ST_ outliers, which is found more often among “ancient” genes.

Our analyses of sequence properties show that the “new” genes do not differ from “ancient” genes in terms of structural properties. This finding seems to be in contrast to previous studies that have identified differences in structural properties between “new” and “ancient” genes (Zhao et al. 2014; Carvunis et al. 2012; Wilson et al. 2017). However, most of these studies focused on annotated genes. Incidentally, one study also found no difference between protein structural properties between “ancient” and “new” genes when they took unannotated ORFs into account in an additional analysis (Carvunis et al. 2012). Other studies employing similar methods to the ones we applied here came to similar conclusions regarding the properties of novel proteins (Schmitz et al. 2018). However, these studies did not compare new genes between populations and as such could only study later steps of emergence compared with this study. The absence of differences in protein structure properties between “new” and “ancient” genes also suggests a high functional potential for new genes as no structural barriers have to be overcome to reach a structurally functional state. The lack of differences in terms of protein structure properties between “new” and “ancient” genes found here also suggests that selection and adaptation of structural properties do not play important roles during the early stages in new gene evolution.

In contrast to protein structural properties, nucleotide sequence properties differ between “new” and “ancient” genes. Differing nucleotide properties would be expected for new genes that emerged from non-coding sequences whose nucleotide sequence properties differ substantially from coding sequences (Wang et al. 2013). Consequently, some of the new genes we found here might have emerged from non-coding sequences (see next section for further support for this hypothesis). Our findings also suggest that there is a step-wise adjustment of the nucleotide sequence properties of young genes over time as they become older genes. However, it is still unclear what the cause of this adjustment is, be it selection for certain nucleotide sequence properties such as GC content or codon usage bias. Recent studies have identified differing GC (Basile et al. 2017) and amino acid content (Basile et al. 2019) as causes of differences in disorder content of eukaryotic proteins.

### Emergence mechanisms of new genes

We searched the sequences of the “new” genes found in our study against the nine-spined stickleback’s genome sequence to determine their mechanism of origin (see “Methods” section). We determined new genes mapping against intergenic regions in the nine-spined stickleback to have emerged de novo (McLysaght and Hurst 2016).

We find 800 of 988 new genes (81%) to have a hit with an E-value ≤10^−3^ in the nine-spined stickleback genome. One hundred and forty-eight (19%) of these genes map against genic regions and 84 (57%) of these gene-mapping genes map against coding sequences. The remaining 652 (of 988 novel) genes map against the nine-spined stickleback’s genome in intergenic regions and, therefore, have likely emerged de novo. This fraction of de novo candidates (66% of new genes) seems quite high, but note that we already filtered out all genes showing any homology to ORFs expressed in the nine-spined stickleback or any other species. Therefore, this ratio is not comparable to ratios found in other studies. Further caveats include the possibility that genes might be expressed specifically in tissues we had no access to and that the (relatively recent) annotation of the nine-spined stickleback might lack some genes. Consequently, results here are likely slightly overestimating the frequency of de novo genes in the three-spined stickleback, i.e., new genes vs. established ones.

Interestingly, we found “new” and “established” genes both to be significantly different from “ancient” genes in terms of genomic measures such as overlap with F_ST_ or CNVs (Fig. 4), hexamer score, and sequence length (both Fig. S5). This finding suggests that some genic properties of emerging genes are markedly different from “ancient” genes and only adapt over long time frames (i.e., hundreds of millions of years) as has been proposed before (Schmitz et al. 2018). This is in contrast to protein properties which we did not find to differ between “new” and “ancient” genes.

## Conclusions

We used multiple transcriptomes from natural populations along a geographic gradient to analyse the expression patterns and properties of new genes and encoded proteins in the three-spined stickleback. The chosen datasets allowed the investigation of dynamics and mode of new gene emergence at the level of populations and therefore at very short evolutionary time scales. By focusing on sequences that neither showed sequence similarity to proteins outside of the analysed species nor sequences being expressed in a sister species, the nine-spined stickleback, we analysed different stages of gene evolution and genetic novelty.

In conclusion, we find 988 new (i.e., three-spined stickleback specific) and 245 established (also present in nine-spined stickleback) genes that have emerged over the course of stickleback evolution (see Fig. 2b).

Most of these younger genes exhibit lower expression levels and were also less broadly expressed across tissues. Younger genes were not as universally expressed across populations and also overlapped with CNVs more often. These findings suggest that younger genes less often encode for essential functions compared with older genes, which preferentially overlap with population-differentiated regions under positive selection. In addition, the findings further stress the importance of analysing weakly expressed transcripts when looking for new genes.

In addition, we find new gene expression to emerge preferentially in gonads and start with relatively low expression levels, gaining higher expression over evolutionary time. We did not find the properties of novel proteins to differ from older proteins. This finding suggests that general order or disorder properties of the encoded proteins do not play a decisive role for many new genes to be retained over longer time scales.

Future studies should investigate how the patterns of new gene emergence found here, i.e. gain of transcription of (presumably intergenic) sequences, relate to their emergence mechanisms, e.g., in the evolution of expression patterns. This information could help infer how frequent the different emergence mechanisms are.

## Methods

### Transcriptome assembly

In this analysis, we used transcriptomes from four tissues: head kidney, spleen, ovaries and testes. Transcriptomes from two immune-related tissues were acquired from a previous study (Huang et al. 2016; accession PRJEB8677 in the European Nucleotide Archive), and from gonads (accession PRJEB26492 in the European Nucleotide Archive). These tissues were derived from the same set of populations and individuals (Supplementary Table 1) whose genomes were sequenced for genome-wide surveys of population differentiation (Chain et al. 2014; Feulner et al. 2015) (accession PRJEB26492 in the European Nucleotide Archive). Raw reads were trimmed using Trimmomatic (Bolger et al. 2014). Trimmed reads were then aligned to the stickleback reference genome (BROADS1 assembly) using HISAT2 (Kim et al. 2015) using default parameters. Transcriptomes for each sample were assembled with StringTie using default arguments (Pertea et al. 2015). The resulting transcriptomes were then merged using the StringTie merge functionality, using the reference annotation (Ensembl68 annotation) as a guide GTF. StringTie was used again on each sample with the merged transcriptome as reference to calculate the per-sample expression strengths. Protein-coding ORFs were predicted using the “transcripts_to_best_scoring_ORFs” script from the PASA suite that employs the TransDecoder algorithm (Haas et al. 2003). Here, we required a minimum protein length of 75 amino acids.

The nine-spined stickleback transcriptome (Guo et al. 2013) was assembled by first trimming the raw reads using Trimmomatic (parameters: ILLUMINACLIP:TruSeq3-SE.fa:3:30:10 LEADING:10 TRAILING:10 SLIDINGWINDOW:4:10 MINLEN:80). Trimmed reads were then assembled using Trinity de novo assembly employing default options (Grabherr et al. 2011).

### New gene detection

Protein age was detected by first blasting all proteins against two outgroup proteomes (mouse, GRCm38; zebra fish, GRCz10). All proteins with hits with an E-value ≤ 10^−3^ were considered “ancient”. In addition, all proteins without hits were searched against the NCBI NR database and all proteins with hits outside of stickleback species were again classified as “ancient”. All proteins were also searched against the Pfam protein domain database (v30.0) and all proteins with hits were classified as “ancient”. The remaining “not ancient” proteins were then searched against the previously assembled nine-spined stickleback’s transcriptome using TBLASTN. Proteins with hits with an E-value ≤ 10^−3^ were then categorised as “established”. All genes that did not have hits in any of these searches were considered “new” (see Fig. 1 and Supplementary Fig. S1).

### Mapping against nine-spined stickleback genome

Protein sequences of new (three-spined stickleback specific) genes were searched against the nine-spined stickleback genome (Varadharajan et al. 2019) using TBLASTN. An E-value of ≤10^−3^ was used as a cut-off for valid hits. For each of the new proteins, the genome annotation at the position overlapping the best hit was analysed. “Gene” or “CDS” annotations were counted as genes or CDS, respectively.

### Analysis of sequence properties

Sequence properties of gene sequence and the encoded proteins were analysed using a number of programs. Disorder was determined using IUPred (Dosztanyi et al. 2005). IUpred was used in the “short” mode and the fraction of amino acids with a disorder prediction above 0.5 was calculated. TANGO (Fernandez-Escamilla et al. 2004) was used to analyse the aggregation propensity of protein sequences. To do this, the fraction of the sequence with an aggregation propensity higher than 5% was determined. CPAT (Wang et al. 2013) was used to calculate hexamer score. The *Danio rerio* model files were used in CPAT.

### Analysis of expression

Expression patterns were analysed based on FPKM. The cut-off for counting absence/presence of expression was set at 1 FPKM. Genes with lower expression levels were still included in other analyses.

Expression specificity was calculated as *τ*. *τ* can vary between 0 and 1, with 0 representing ubiquitously expressed genes and 1 specifically expressed genes (Yanai et al. 2004). *τ* is calculated as follows:

$$\tau =\frac{\mathop{\sum }\nolimits_{i=1}^{N}1-{x}_{i}}{N-1},$$

where *N* represents the number of tissues and *x*_*i*_ a normalised expression value in a tissue.

### Analysis of F_ST_ and CNVs

F_ST_ measurements were acquired from a genome-wide divergence analysis between the same population pairs of sticklebacks as used in this study (Feulner et al. 2015). CNV regions were established in a previous study that evaluated read depth across the genome of these stickleback individuals (Chain et al. 2014). For the analysis of CNVs, only CNVs present in at least five individuals, but <50% of the individuals were considered. This was done because CNVs present in most of the analysed transcriptomes might represent a mutation in the reference genome. In addition, at least 25% of a gene’s sequence was required to overlap with a CNV to be considered as overlapping.

### Analysis of CpG depletion

CpG depletion is a measure for gene-body methylation since an absence of CpG dinucleotides hints at the presence of previous methylation. This is because methylated CpG dinucleotides mutate with a higher likelihood than non-methylated CpG dinucleotides. Here, CpG depletion was determined by calculating the ratio of GC dinucleotides over the product of the frequencies of G and C nucleotides:

$$D=\frac{{f}_{\rm{GC}}}{{f}_{\rm{G}}{f}_{\rm{C}}},$$

here *f*_GC_ is the observed fraction of CpG dinucleotides, while *f*_G_*f*_C_ is the expected fraction.

For each gene, the CpG depletion for all combined exons was calculated. In addition, the median CpG depletion of all intergenic sequences was calculated.

## Supplementary information

Supplementary Figures

Supplementary Table 1

## Data Availability

The transcriptomic data analysed in this study can be found under the accessions PRJEB8677 and PRJEB26492 in the European Nucleotide Archive (see also Supplementary Table S1). Transcript sequences, expression values and calculated transcript ages as well as scripts are available under 10.6084/m9.figshare.11889771.
