# Supplementary material for: Evolution of novel genes in three-spined stickleback populations
Source: Heredity (Edinb). 2020 Jun 4;125(1-2):50–9. doi: 10.1038/s41437-020-0319-7 (PMC7413265; doi:10.1038/s41437-020-0319-7)
Supplement: Supplementary file 1 — Supplementary Figures [file 41437_2020_319_MOESM1_ESM.pdf]

Supplementary Materials & Methods

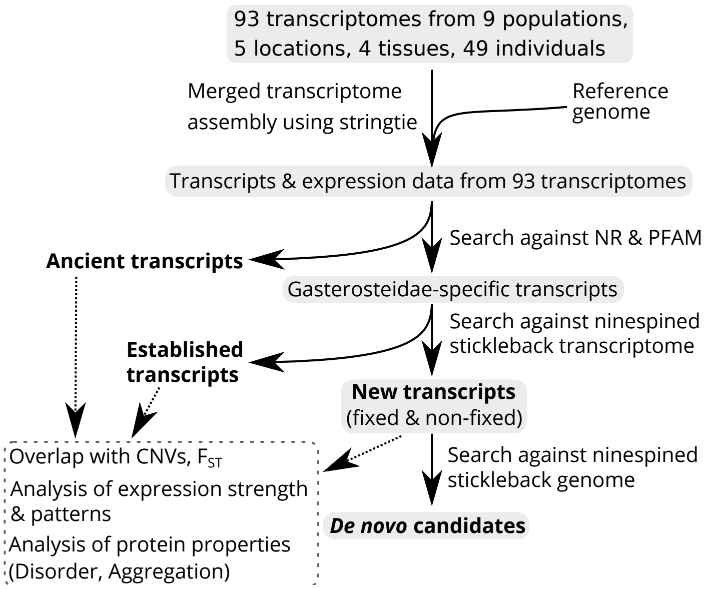

Figure S 1. Flowchart describing how genes classes were derived here.

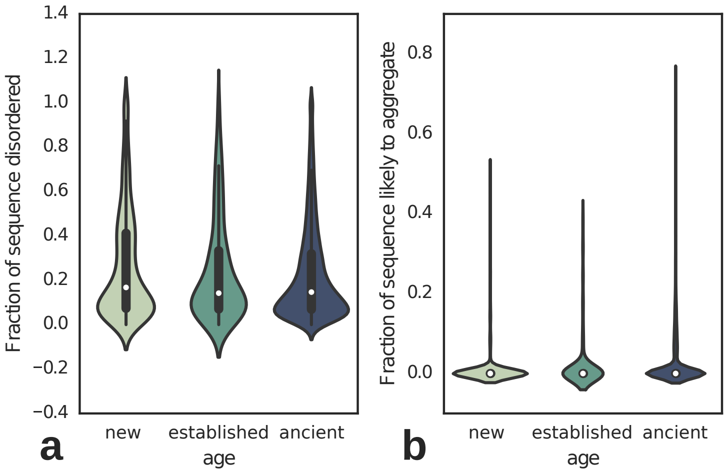

Figure S 2. Sequence properties of proteins and their coding DNA sequences separated by age classes. **a**: Violin plot of intrinsic disorder as calculated with IUPred (short). **b**: Violin plot of aggregation propensity as calculated with TANGO.

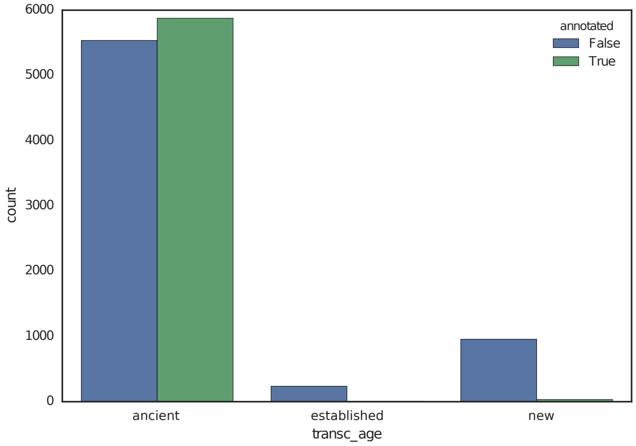

Figure S 3. Amount of genes annotated per age group.

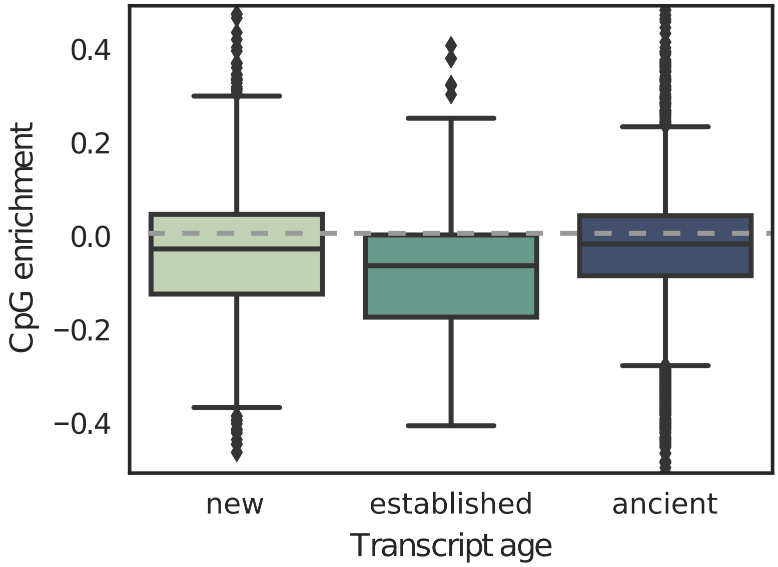

Figure S 4. CpG enrichment in the three stickleback genes by age group. Only exonic sequences were analysed. Grey bar represents the intergenic CpG enrichment level.

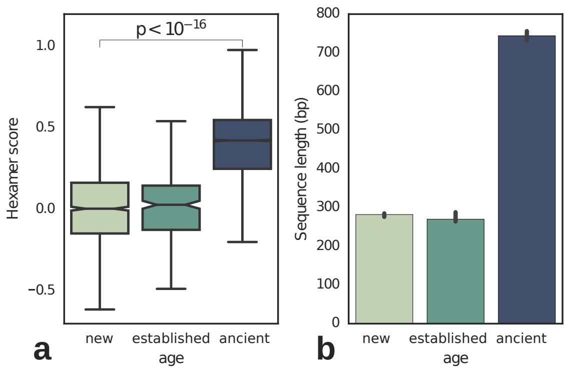

Figure S 5. Sequence properties of proteins and their coding DNA sequences separated by age class. **a**: Box plot of hexamer score as calculated with CPAT. **b**: Bar plot of median sequence length in nucleotides. Also shown is the 95% confidence interval of 1000 bootstraps as the "candle wick".

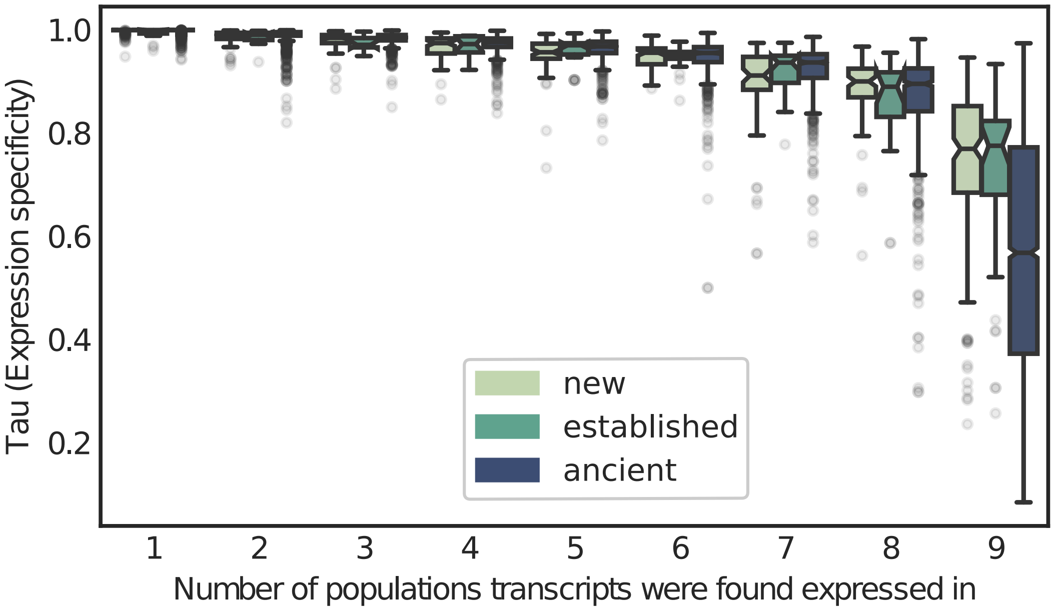

Figure S 6. Box plot of expression specificity (Tau) of genes by the number of populations the gene was found expressed in. Outliers are not shown.

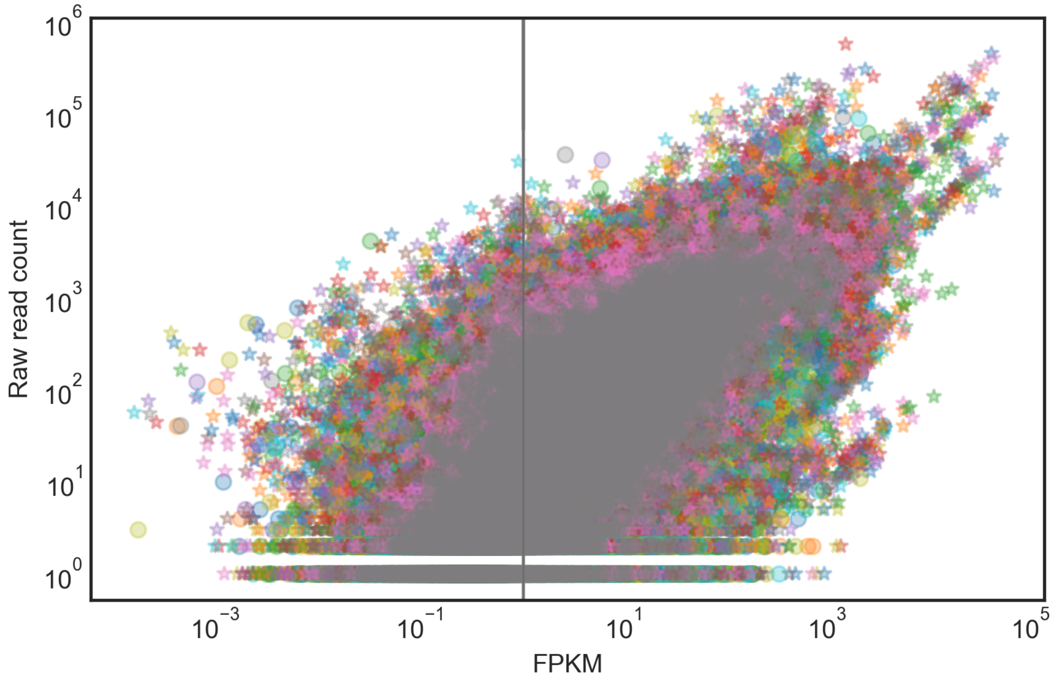

Figure S 7. Scatter plot of FPKM vs. read count for all transcriptomes. Immune tissue samples are marked with a star symbol, gonad tissues with a circle.

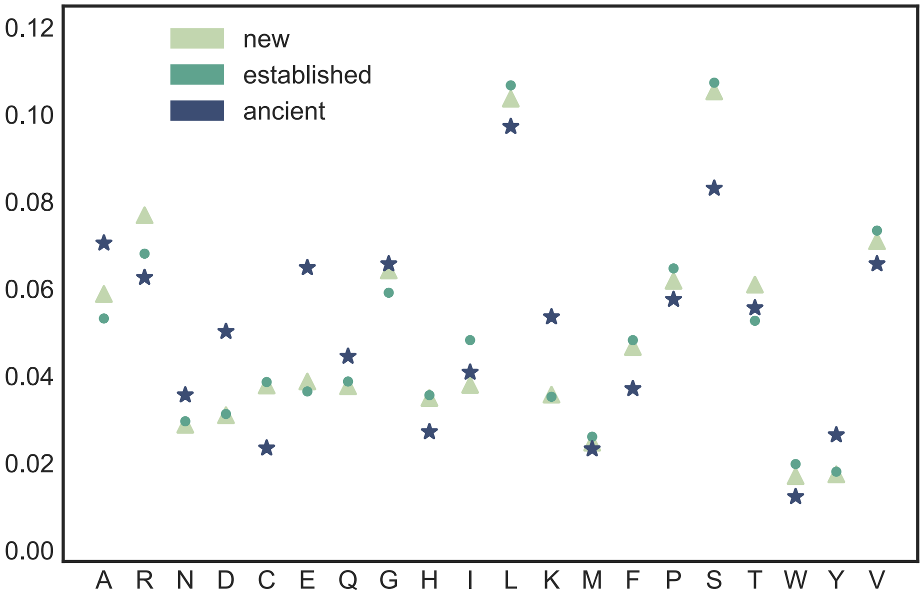

Figure S 8. Relative frequencies of amino acids in the sequences of coding sequences of the different age categories.
